# Transfusions and blood loss in total hip and knee arthroplasty: a prospective observational study

**DOI:** 10.1186/s13018-015-0188-6

**Published:** 2015-03-28

**Authors:** Malin S Carling, Anders Jeppsson, Bengt I Eriksson, Helena Brisby

**Affiliations:** Department of Orthopaedics, Sahlgrenska University Hospital, Gothenburg, SE 413 45 Sweden; Department of Orthopaedics, Institute of Clinical Sciences, Sahlgrenska Academy, University of Gothenburg, Gothenburg, Sweden; Department of Cardiothoracic Surgery, Sahlgrenska University Hospital, Gothenburg, Sweden; Department of Molecular and Clinical Medicine, Institute of Medicine, Sahlgrenska Academy, University of Gothenburg, Gothenburg, Sweden

**Keywords:** Arthroplasty, Hemorrhage, Transfusion

## Abstract

**Background:**

There is a high prevalence of blood product transfusions in orthopedic surgery. The reported prevalence of red blood cell transfusions in unselected patients undergoing hip or knee replacement varies between 21% and 70%. We determined current blood loss and transfusion prevalence in total hip and knee arthroplasty when tranexamic acid was used as a routine prophylaxis, and further investigated potential predictors for excessive blood loss and transfusion requirement.

**Methods/materials:**

In total, 193 consecutive patients undergoing unilateral hip (*n* = 114) or knee arthroplasty (*n* = 79) were included in a prospective observational study. Estimated perioperative blood loss was calculated and transfusions of allogeneic blood products registered and related to patient characteristics and perioperative variables.

**Results:**

Overall transfusion rate was 16% (18% in hip patients and 11% in knee patients, *p* = 0.19). Median estimated blood loss was significantly higher in hip patients (984 vs 789 mL, *p* < 0.001). Preoperative hemoglobin concentration was the only independent predictor of red blood cell transfusion in hip patients while low hemoglobin concentration, body mass index, and operation time were independent predictors for red blood cell transfusion in knee patients.

**Conclusions:**

The prevalence of red blood cell transfusion was lower than previously reported in unselected total hip or knee arthroplasty patients. Routine use of tranexamic acid may have contributed. Low preoperative hemoglobin levels, low body mass index, and long operation increase the risk for red blood cell transfusion.

## Introduction

Orthopedic surgery, especially spine surgery and arthroplasty surgery, is associated with excessive bleeding and a high demand for blood transfusion [1,2]. In routine total hip arthroplasty and total knee arthroplasty, the prevalence of allogeneic red blood cell (RBC) transfusions has been reported to be between 21% and 70%, with the majority of authors reporting figures in the middle of the range [1,3-5].

Allogeneic RBC transfusions are sometimes lifesaving, but also associated with a number of risks, including transfusion-related lung injury (TRALI), immunomodulation, and transmission of pathogens [6,7]. Studies have also indicated that blood transfusion in itself may increase the risk of early and late morbidity and mortality [8,9]. Extensive perioperative bleeding is associated with an increased transfusion rate, as anticipated. However, other patient-related and surgery-associated factors, such as gender, preoperative hemoglobin levels, and operative technique are also predictive of transfusion requirement in different surgical settings [2,10,11]. There is sparse prospective data on risk factors for excessive bleeding and transfusion of blood products in total hip and knee arthroplasty.

In the present study, we assessed blood loss and transfusion requirements after elective primary total hip and knee arthroplasty when tranexamic acid was used as a routine prophylaxis, and we aimed at identifying clinically relevant predictors of excessive blood loss and transfusion requirements.

## Materials and methods

### Study design

Patients with osteoarthritis undergoing total hip or knee arthroplasty were included in a prospective observational study where transfusions and blood loss were registered and related to perioperative characteristics.

### Patients and settings

A total of 193 consecutive patients (mean age 67 ± 11 years, 49% women), with the diagnosis osteoarthritis undergoing unilateral primary hip (*n* = 114) or knee (*n* = 79) arthroplasty at two centers (Sahlgrenska University Hospital and Kungälv Hospital), were included in the study between October 2009 and January 2011. Exclusion criteria were known liver disease or coagulation disorder.

### Ethics, consent, and permissions

The Regional Ethical Review Board in Gothenburg approved the study (date of approval 11 September 2009, reference number 072–08). All patients gave informed written consent before inclusion in the study.

### Clinical management

Spinal anesthesia was used in 160 (83%) of the patients (95 hip, 65 knee) and general anesthesia in the remaining patients. Dabigatran was used as thrombosis prophylaxis in 179 patients (104 hip, 75 knee) while 14 patients received low molecular weight heparin (LMWH) (15 hip, 4 knee). The first dose of dabigatran was administered 1–4 h after surgery while the first dose of LMWH was administered in the evening before surgery. All patients received tranexamic acid (Cyklokapron®; Pfizer, Sollentuna, Sweden), 10 mg/kg body weight; for hip patients, one dose after start of anesthesia and one dose 3 h later; for knee patients, one dose 10–15 min before release of the tourniquet and one dose 3 h later, according to the local routine.

All patients in the knee group received cemented prostheses and they were operated with a tourniquet. In the hip group, 55 patients received cemented prostheses, 25 received uncemented prostheses, 9 received hybrid arthroplasties (uncemented acetabular component and cemented femoral component), and 10 received reverse hybrid arthroplasty (cemented acetabular component and uncemented femoral component). None of the knee or hip patients had wound drainage postoperatively.

The following baseline variables were recorded: age; gender; BMI; medication prior to surgery, including the use of acetylsalicylic acid (ASA), nonsteroidal anti-inflammatory drugs (NSAIDs), or selective serotonin receptor inhibitors (SSRIs); type of surgery; and thrombosis prophylaxis. Patients on ASA or NSAIDs were urged to discontinue these medications 3 days before surgery. Medication with potent platelet inhibitors, such as clopidogrel, were stopped at least 1 week before surgery. Blood samples from a peripheral vein for hemoglobin (Hb), platelet count, activated partial thromboplastin time (aPTT), and prothrombin time (PT) analyses were obtained <24 h before surgery. Hemoglobin level was also measured 24–48 h postoperatively in order to calculate blood loss.

The following perioperative variables were recorded: duration of operation, bleeding during surgery (intraoperatively), transfusion requirements intraoperatively, and postoperatively until discharge or until reoperation and autologous transfusion of wound blood after cell saver processing. Intraoperative bleeding was calculated from blood retrieved from wound suction plus the estimated amount of blood in the swabs.

### Analyses

Whole blood samples for determination of Hb and platelet count analyses were collected in K2 EDTA plastic tubes (1.8 g/L EDTA; BD Vacutainer, Plymouth, UK). For PT and APTT analyses, blood samples were collected in citrated plastic tubes (2.7 mL 0.129 mmol/L sodium citrate; BD Vacutainer, Plymouth, UK). Hb, platelet count, aPTT, and PT were analyzed with clinical standard methods at the accredited local hospital laboratories. PT is reported as the international normalized ratio (INR); reference value: <1.2. The reference value for APTT is 30–42 s.

### Definitions and formulae

Estimated blood loss (EBL) was calculated based on the drop in Hb between the preoperative measurements and the measurements 24–48 h postoperatively according to a formula developed by Brecher [12]. Only intraoperative transfusions are included in the calculation since none of the patients received postoperative transfusion in the interval between end of surgery and before the blood sample taken 24–48 h postoperatively for Hb determination. The formula used was as follows: EBL = ((“estimated blood volume (EBV)” × “hematocrit preoperatively” − EBV × “hematocrit postoperatively”) + (“intraoperative RBC transfusion” × 200 + “intraoperative Cellsaver transfusion” × 0.55)) / 0.35 [12].

For EBV, the formula is as follows: EBV = (0.0235 × height in cm^0.42246 × weight in kg^0.51456) × *k*, where *k* = 2,430 for women and 2,530 for men. Patients in the upper 75th percentile of EBL/kg in the hip and knee group, respectively, were defined as excessive bleeders while all other patients were considered to be non-excessive bleeders.

The study population was also divided into a transfusion group with those patients who received allogeneic RBC transfusions until discharge from hospital and a no-transfusion group with the patients who did not receive any allogeneic RBC transfusion. No pre-specified transfusion criteria were used during the study period, i.e., transfusions were prescribed at the discretion of the attending physician.

### Statistics

Continuous variables were compared with Student *t*-test or Mann-Whitney *U* test, and categorical variables were compared with Chi-square test. In the statistical analysis, PT was considered a continuous variable. Statistical significance was defined as a *p* value of <0.05. Statistical models for identification of predictors of blood loss and transfusion were made with logistic regression analysis. For multiple regression analysis, stepwise logistic regression analysis was used. The following variables were included in the analysis: age; gender; BMI; use of NSAIDs, ASA, or SSRI before surgery; type of anesthesia; operation time; and preoperative Hb, platelet count, PT, and aPTT. For analyses of RBC transfusions, weight was also included. For statistical calculations, IBM SPSS 20.0 software was used.

## Results

### General

All patients survived the perioperative period, and there were no major complications except one hip patient that was re-operated because of inappropriate stem placement, and one knee patient was re-operated because of early, deep infection. There were no significant differences in any of the registered parameters between the two study hospitals. Patients in the knee group had a significantly higher BMI than patients in the hip group (*p* < 0.001). Operation time was significantly longer for the hip group than for the knee group (*p* = 0.004). For patient characteristics and perioperative variables (Table 1).

**Table 1:** **Patient characteristics**

	**Hip arthroplasty**	**Knee arthroplasty**	***p*** **value**
	***n*** = **114**	***n*** = **79**	**(hip vs. knee)**
**Male gender**	62 (54%)	32 (41%)	0.058
**Preop medication**			
**NSAID**	30 (26%)	16 (20%)	0.33
**Acetylsalicylic acid**	18 (16%)	18 (23%)	0.22
**SSRI**	10 (9%)	9 (11%)	0.55
**Thromboprophylaxis**			
**Dabigatran**	104 (91%)	75 (95%)	0.33
**LMWH**	10 (9%)	4 (5%)	
**Anesthesia**			
**Spinal**	95 (83%)	65 (82%)	0.85
**General**	19 (17%)	14 (18%)	
**BMI** ** (kg/** **m** ^**2**^ **)**	27 ± 4.1 (17–40)	30 ± 4.5 (22–49)	*<0.001*
**Age** ** (years)**	66 ± 12	69 ± 10	0.086
**Hemoglobin** ** (g/** **L)**	136 ± 13	133 ± 12	0.24
**Platelet count** ** (10** ^**9**^ **/L)**	283 ± 65	280 ± 72	0.81
**PT** ** (INR)**	1.0 ± 0.1	1.0 ± 0.1	0.77
**aPTT** ** (s)**	34 ± 3.8	33 ± 3.3	0.57
**Operation time** ** (min)**	125 ± 28	113 ± 28	*0.004*
**RBC transfusion**	21 (18%)	9 (11%)	0.18
**RBC transfusion** ** (units)**	0 (0–4)	0 (0–3)	0.22
**EBL** ** (mL)**	984 (0–4571)	789 (0–1927)	*0.001*
**EBL/** **kg (** **mL** **/kg)**	12 (0–33)	9 (0–23)	*<0.001*
**Cellsaver (** **mL)**	0 (0–1200)	0	*0.007*

*Abbreviations*: *aPTT* activated partial thromboplastin time, *BMI* body mass index, *EBL* estimated blood loss, *LMWH* low molecular weight heparin, *NSAID* nonsteroidal anti-inflammatory drugs, *PT* prothrombine time, *RBC transfusion* red blood cell transfusion, *SSRI* selective seretonine receptor inhibitor.

Mean and standard deviation**,** median and range**,** or number and percentage.

### Bleeding and estimated blood loss

**Figure 1:**
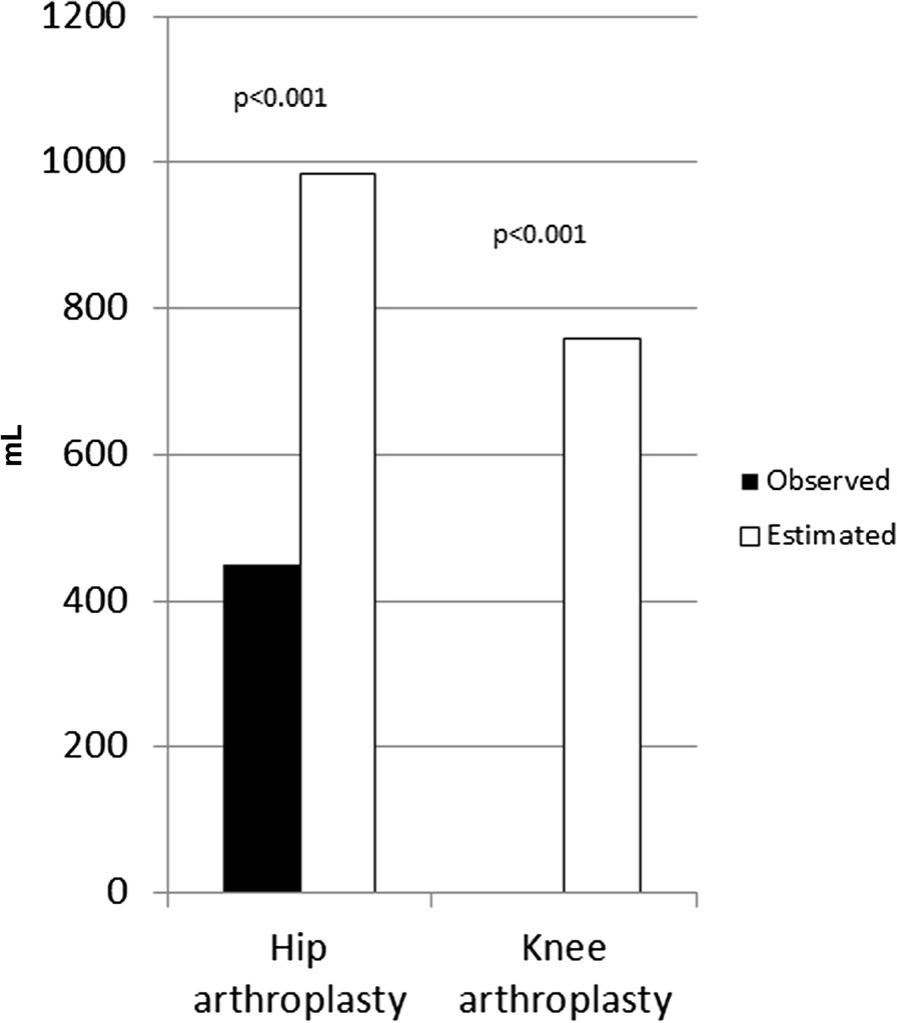
**Observed and estimated median perioperative bleeding in patients undergoing total hip arthroplasty or total knee arthroplasty.**

Observed median intraoperative bleeding for hip patients was 450 mL (range 150–3,000) and for knee patients 0 mL (range 0–600). The median EBL was 984 and 789 mL for hip and knee patients, respectively. There was a significant difference between observed perioperative bleeding and EBL for both hip and knee patients (Figure 1). There was a significant difference in median EBL (*p* = 0.001) and EBL/kg (*p* < 0.001) between the hip group and the knee group (Table 1).

In hip patients, a univariate logistic regression analysis showed that female gender, low BMI, and long operation time were associated with EBL/kg > 75th percentile (>17.1 mL/kg) (Table 2). In the multiple regression analysis, female gender (odds ratio (OR) 3.91 (95% confidence interval 1.41–10.88) per kg, *p* = 0.009), low BMI (OR 0.82 (0.71–0.94) per unit, *p* = 0.005), and long operation time (OR 1.03 (1.01–1.05) per min, *p* = 0.04) increased the risk of excessive blood loss.

**Table 2:** **Univariate risk factor analysis in total hip arthroplasty patients for excessive bleeding and transfusions**

	**Excessive bleeding** ** (EBL/** **kg >** **17.1 mL/** **kg)**			**Allogeneic RBC transfusion**		
	**OR**	**95% ** **CI**	***p*** **value**	**OR**	**95% ** **CI**	***p*** **value**
**Age** ** (years)**	1.00	0.99–1.07	0.11	1.05	1.01–1.10	*0.022*
**Sex** ** (F)**	3.39	1.37–8.38	*0.008*	7.04	2.19–22.63	*0.001*
**BMI** ** (kg/** **m** ^**2)**^	0.83	0.73–0.95	*0.006*	0.90	0.79–1.03	0.13
**Weight** ** (kg)**	0.92	0.89–0.96	*<0.001*	0.94	0.91–0.98	*0.003*
**NSAID** ** (No)**	1.42	0.51–3.93	0.50	0.66	0.24–1.83	0.42
**ASA** ** (No)**	1.17	0.35–3.89	0.80	0.75	0.22–2.57	0.65
**SSRI** ** (No)**	0.45	0.12–1.73	0.24	0.29	0.08–1.15	0.079
**Hemoglobin** ** (g/** **L)**	0.97	0.94–1.01	0.12	0.87	0.82–0.93	*<0.001*
**Platelet count** ** (10** ^**9**^ **/L)**	1.01	1.00–1.01	0.17	1.00	1.00–1.01	0.52
**PT** ** (INR)**	0.76	0.001–70.9	0.90	0.03	0.00–7.71	0.21
**aPTT** ** (s)**	1.01	0.90–1.13	0.87	1.05	0.93–1.17	0.45
**Operation time** ** (minutes)**	1.02	1.00–1.04	*0.017*	1.02	1.00–1.03	0.065
**Anesthesia** ** (spinal)**	0.89	0.29–2.75	0.85	0.82	0.24–2.77	0.75
**EBL/** **kg**		n.a.		1.17	1.09–1.26	*<0.001*

*Abbreviations*: *aPTT* activated partial thromboplastin time, *BMI* body mass index, *EBL* estimated blood loss, *LMWH* low molecular weight heparin, *NSAID* nonsteroidal anti-inflammatory drugs, *PT* prothrombine time, *RBC transfusion* red blood cell transfusion, *SSRI* selective seretonine receptor inhibitor.

In knee patients, low BMI and high preoperative Hb were univariately associated with EBL/kg > 75th percentile (>12.7 mL/kg) (Table 3). In the multiple regression analysis, low BMI (OR 0.77 (0.64–0.93) per unit, *p* = 0.006) and high preoperative Hb (OR 1.11 (1.04–1.17) per g/L, *p* = 0.001) increased the risk of excessive bleeding.

**Table 3:** **Univariate risk factor analysis in total knee arthroplasty patients for excessive bleeding and transfusions**

	**Excessive bleeding** ** (EBL/** **kg >** **12.7 mL/** **kg)**			**Allogeneic RBC transfusion**		
	**OR**	**95% ** **CI**	***p*** **value**	**OR**	**95% ** **CI**	***p*** **value**
**Age** ** (years)**	1.02	0.97–1.08	0.45	0.99	0.92–1.06	0.79
**Sex** **(F)**	0.69	0.24–1.95	0.49	0.29	0.068–1.28	0.10
**BMI** ** (kg/** **m** ^**2**^ **)**	0.81	0.69–0.95	*0.010*	0.78	0.62–0.98	*0.034*
**Weight** ** (kg)**	0.96	0.92–1.0	*0.046*	0.97	0.92–1.02	0.26
**NSAID** ** (No)**	0.94	0.26–3.34	0.92	0.88	0.16–4.68	0.88
**ASA** ** (No)**	1.14	0.32–4.0	0.84	2.57	0.3–22.02	0.39
**SSRI** ** (No)**	1.12	0.21–5.93	0.89	1.03	0.11–9.37	0.98
**Hemoglobin** ** (g/** **L)**	1.09	1.03–1.16	*0.001*	0.91	0.84–0.97	*0.007*
**Platelet count** ** (10** ^**9**^ **/L)**	1.0	0.99–1.01	0.56	1.001	0.99–1.01	0.88
**PT** ** (INR)**	0.001	0.00–1.03	0.051	0.036	0–220.4	0.46
**aPTT** ** (s)**	0.91	0.78–1.07	0.26	1.34	1.03–1.74	*0.028*
**Operation time** ** (minutes)**	1.00	0.98–1.02	0.87	1.03	1.00–1.06	*0.034*
**Anesthesia** ** (spinal)**	2.12	0.43–10.48	0.35	0.72	0.13–3.92	0.71
**EBL/** **kg**		n.a.		1.020	0.88–1.18	0.795

*Abbreviations*: *aPTT* activated partial thromboplastin time, *ASA* acetyl salicylic acid, *BMI* body mass index, *EBL* estimated blood loss, *LMWH* low molecular weight heparin, *NSAID* nonsteroidal anti-inflammatory drugs, *PT* prothrombine time, *RBC transfusion* red blood cell transfusion, *SSRI* selective seretonine receptor inhibitor.

### Transfusions

Ten patients, all in the hip group, received intraoperative re-transfusion of cell saver processed wound blood. The median re-transfused volume was 300 mL (range 150–1,200 mL). None of these patients received allogeneic RBC transfusion.

In total, 30 patients (16%) received allogeneic blood transfusions during their hospital stay, 21 in the hip group and 9 in the knee group. There was no statistically significant difference in transfusion prevalence between the hip group and the knee group (18% vs. 11%, *p* = 0.19). One patient received transfusion with fresh frozen plasma intraoperatively. Thirteen patients, all in the hip group, received allogeneic RBC transfusion intraoperatively, while none of the knee patients received intraoperative transfusion. Eighteen patients received allogeneic RBC transfusion more than 24 h postoperatively, 8 in the hip group and 10 in the knee group. One patient in the hip group received both intraoperative and postoperative allogeneic RBC transfusion.

In hip patients, low preoperative Hb, high age, female gender, and large EBL/kg were univariately associated with perioperative allogeneic RBC transfusion (Table 2). In multiple regression analyses, preoperative Hb was the only factor significantly predictive of allogeneic RBC transfusion (OR 0.87 (0.82–0.93) per g/L, *p* < 0.001).

In knee patients, low preoperative Hb, low BMI, long aPTT, and long operation time were univariately associated with allogeneic RBC transfusion (Table 3). In multiple regression analysis, low BMI (OR 0.71 (0.51–0.98) per unit, *p* = 0.034), low preoperative Hb (OR 0.71 (0.51–0.98) per g/L, *p* = 0.015), and long operation time (OR 1.04 (1.00–1.07) per minute, *p* = 0.036),increased the risk of RBC transfusion.

## Discussion

The main findings in the present study were as follows: (1) The rate of transfusion (16%) was lower than previously reported in unselected patients undergoing total primary hip and knee arthroplasty. (2) The lower transfusion rate was not accompanied with any indications of increased complication rate. (3) Preoperative hemoglobin concentration, body mass index, and operating time were the most important risk factors for excessive blood loss and transfusion.

In the present study, the perioperative estimated blood loss volume was greater for hip arthroplasty patients than for knee arthroplasty patients (Table 2), which contrasts with the results of a previous study [13]. However, the difference was limited (median difference 195 mL, or 3.1 mL/kg) and may not be clinically relevant. The results also show that even though knee arthroplasty is performed with a tourniquet and the intraoperative bleeding is minimal, the patients have a significant amount of perioperative blood loss. Median EBL in knee patients was 790 mL (range 0–1,927) in the present study, and even though this is an estimated volume, it reflects a blood loss that may affect the patients. Hidden blood loss after knee arthroplasty with a tourniquet is a known concern, and several studies have shown that there is no difference in total perioperative bleeding after knee arthroplasty with or without a tourniquet [14,15]. In the hip patients, in the present study, also a relatively large hidden blood loss was calculated and may also be a concern. This is in accordance with the findings in a study by Liu et al. [16].

Low BMI was associated with an increased risk of excessive blood loss. This is in accordance with findings from heart surgery, where low BMI has been shown to be a risk for reoperation because of excessive bleeding [17]. For hip and knee arthroplasty, the results vary in different studies. In studies by Prasad et al. and Hrnack et al., BMI was not found to be associated with blood loss in neither hip nor knee patients [18,19]. For hip arthroplasty patients, Bowditch et al. reported an increased risk of bleeding in obese patients [20]. The contrasting findings in different studies regarding the association between BMI and bleeding may be at least partially explained by the fact that some studies have used bleeding volume *per se* and others have used bleeding per kg in the calculations. The finding of increased risk of bleeding with longer operation time may be a direct effect of time, if a relatively constant amount of bleeding per time unit can be expected. However, it may also be the other way around; a higher degree of intraoperative bleeding may extend the surgical time. With the present data, we cannot distinguish between these mechanisms.

In contrast to the blood loss results, there was no statistically significant difference in transfusion requirements between hip and knee patients (*p* = 0.19). However, there was a numerical difference with higher transfusion requirements in hip patients (18% vs. 11%), and the possibility of statistical type-II error cannot be excluded. The total RBC transfusion requirements for unselected hip and knee arthroplasty patients was 16%, 30 patients out of 194, which is markedly lower than in most previous publications [2,4,21-23]. Borghi et al. reported allogeneic RBC transfusion requirements of only 10% for total hip and knee arthroplasty patients [24]. However, that study and other studies reporting low transfusion rates have been focused studies, investigating the use of a new transfusion protocol [25,26] or the use of autologous transfusion programs [27]. It is difficult to compare studies since, besides differences in study design and study populations, some investigators only included postoperative transfusions but not intraoperative ones in their analysis. In the present study, we report the total number of perioperative transfusion. No specific transfusion protocol or transfusion triggers were used, and a large number of physicians were involved in the care of these patients.

In accordance to previous studies [2,28-30], we found that a low preoperative Hb was a significant risk factor for receiving allogeneic RBC transfusions. This is not surprising, since a low Hb is a known transfusion trigger. Studies on anemic patients undergoing major orthopedic surgery have shown that optimizing the patients’ Hb preoperatively with erythropoietin and iron treatment may reduce transfusion rates [31].

A number of different measures can be taken to minimize the need for allogeneic transfusions, including transfusions of predonated autologous blood, cell salvage systems, and administration of pro-haemostatic drugs. It has been demonstrated that predonated autologous blood transfusions are cost-effective and reduce allogeneic blood transfusions in selected patient groups [32]. The use of autotransfusion, or cell salvage, reduces the number of intraoperative allogeneic RBC transfusions [33]. This was also noted in the present study where none of the ten hip patients who received autotransfusion of cell-salvaged blood did require transfusion of allogeneic blood. The introduction of new transfusion protocols and policies may also affect the rates of transfusion [34].

Tranexamic acid is commonly used in hip and knee arthroplasty surgery in Sweden. The use of tranexamic acid and other anti-fibrinolytic substances is well-studied and known to significantly decrease the number of transfusions in cardiac and orthopedic surgery [35]. Our study population received tranexamic acid according to hospital guidelines, and this may have contributed to low bleeding volumes and low transfusion rates.

One limitation of the present study was the restricted number of patients included, which did not allow further subgroup analysis. The lack of a specific transfusion protocol or transfusion triggers as well as the involvement of a large number of physicians involved in the care of these patients could also possibly have influenced the results. However, one of the strengths of this study is that the investigation was performed in consecutive patients in an everyday clinical setting without selection bias.

## Conclusions

This study showed that an unselected group of patients undergoing elective primary total hip or knee arthroplasty in an everyday clinical setting had a lower risk of receiving a blood transfusion as compared to many reports in the literature. Predictors for receiving allogeneic transfusion were a low preoperative Hb and a low BMI. With increased awareness in the organization and structured transfusion programs, the transfusion rate may be possible to reduce further.
